# The Evidence for Fertility Preservation in Pediatric Klinefelter Syndrome

**DOI:** 10.3389/frph.2021.629179

**Published:** 2021-08-03

**Authors:** Celina J. Pook, Alessandra Cocca, Anna Grandone, Mohamed Al-Hussini, Wayne Lam

**Affiliations:** ^1^Department of Urology, Guy's and St Thomas' National Health Service (NHS) Foundation Trust, London, United Kingdom; ^2^GKT School of Medical Education, King's College London, London, United Kingdom; ^3^Paediatric Endocrine and Diabetes Department, Evelina London Children's Hospital, Guy's and St Thomas' National Health Service (NHS) Foundation Trust, London, United Kingdom; ^4^Department of Woman, Child, General and Specialized Surgery, Università degli Studi della Campania Luigi Vanvitelli, Naples, Italy; ^5^Division of Urology, Department of Surgery, Queen Mary Hospital, The University of Hong Kong, Hong Kong, China

**Keywords:** fertility preservation, Klinefelter syndrome, microdissection testicular sperm extraction, testosterone replacement therapy, pediatric fertility preservation

## Abstract

Klinefelter syndrome (KS) is a common cause of non-obstructive azoospermia (NOA). Advances in fertility preservation (FP) techniques, such as the use of microdissection testicular sperm extraction (micro-TESE), have improved sperm retrieval rates (SRR) up to 40–50% in this population. Age has been suggested to have an impact on FP, postulating that sperm production may deteriorate over time due to germ cell loss. As such, sperm retrieval for patients with KS at a younger age has been proposed to further improve SRR; however, whether such practice pragmatically improves SRR is yet to be determined, and controversy remains with concerns over trauma caused by FP procedures on further impairment of testicular function. There has also been a debate on the ethics of performing FP procedures in the pediatric population. Optimizing FP for patients with KS invariably requires a holistic multidisciplinary approach. This review aimed to evaluate the latest evidence in performing FP in pediatric patients with KS, and discuss the controversy surrounding such practice. Hormonal changes in patients with KS during childhood and the use of hormonal manipulation to optimize SSR in this population have also been reviewed.

## Introduction

Klinefelter Syndrome (KS) is a chromosomal disorder in which there is at least one extra X chromosome when compared with the normal male karyotype of 46, XY. It has an estimated prevalence of 1 in 500–600 males, with approximately two-thirds diagnosed in adulthood at a mean age of 27.5 years (1). Clinical manifestation of KS can be widely variable. Some patients present with NOA infertility or hypogonadotrophic hypogonadism, whilst others present with associated features including tall stature, narrow shoulders, decreased facial hair, small testes, and gynaecomastia (2); however, some patients with mosaic KS often demonstrate very few clinical signs or symptoms and appear phenotypically normal.

Klinefelter Syndrome is the most common chromosomal anomaly found in male patients with non-obstructive azoospermia (NOA), accounting for ~10% of all patients with azoospermia (3). As such, many patients are identified to have KS during investigations for male-factor infertility in later adult life; however, with improved recognition of pediatric symptoms, growing use of non-invasive prenatal screening and higher accessibility of quantitative fluorescent PCR (QR-PCR), the number of patients with KS diagnosed during childhood has increased, with the developmental delay being the commonest presenting complaint (4).

The current focus of pediatric KS treatment surrounds targeting endocrine deficits to improve growth, muscle mass, and prevent delayed puberty. Recent investigations in patients with KS and NOA have demonstrated a time-sensitive pattern of germ cell depletion, suggesting younger age is a potential positive predictor of success in sperm retrieval (5). The role of FP prior to adulthood to optimize sperm retrieval rates (SRR) in patients with KS has drawn immense interests in recent studies examining the role of hormone replacement therapy in optimizing intratesticular hormonal environment in patients with KS, and other studies exploring FP techniques to optimize identification of areas with healthy testicular seminiferous tubules and to minimize testicular insult during sperm harvest, presenting outcomes in terms of sperm retrieval and pregnancy rates. These developments raise discussions surrounding the ethical basis for FP in a pediatric setting and underpin the importance of a multidisciplinary team approach. This review presents the latest evidence for/against fertility preservation (FP) in the pediatric population with KS.

## Physiological and Endocrinological Changes in KS

The endocrinology of men with KS is typically recognized by a hormone profile of high serum follicle-stimulating hormone (FSH), low luteinizing hormone (LH), and low testosterone levels; however, various significant changes occur from birth to adulthood that impacts spermatogenesis in patients with KS.

During early development in males with KS, the first peak of testosterone occurs at “mini-puberty,” which involves the activation of the hypothalamus-pituitary-gonadal axis within the first 3–6 months of life. During this time, testosterone, FSH, and LH levels can reach pubertal or even adult levels; however, there is contrasting evidence on whether testosterone levels are comparatively lower inpatients with KS during mini-puberty to healthy males, suggesting a role of Leydig cell dysfunction occurring early in childhood (6). During the pre-pubertal period, patients with KS often have normal levels of testosterone, FSH, LH, and inhibin B (7), but a reduced number of spermatogonia. As puberty begins, LH and FSH levels start to rise above normal levels, whilst testosterone levels decline; however, testosterone levels can still be within the normal range in up to 60% of patients with KS during puberty, adequate for reaching appropriate virilization. Though a state of relative androgen insufficiency may be present, leading to the development of various signs, such as gynecomastia (8, 9). As a result, testosterone replacement therapy is not needed for puberty induction but is often started where signs of hypogonadism or biochemical evidence of testosterone deficiency are present (10). Reduction in the number of germ cells, hyalinization of the tubules, degeneration of Sertoli cells, and hyperplasia of Leydig cells are also common during puberty in patients with KS (11).

## Sperm Retrieval

Males with NOA may harbor small, patchy foci of productive seminiferous tubules capable of spermatogenesis, including KS testes (12, 13). Possible explanations for this include the following: (1) some XXY spermatogonial stem cells (SSC) can complete meiosis and produce functional spermatozoa, as illustrated in a previous fluorescence *in-situ* hybridization (FISH) study in 10 patients with KS (14) and (2) a small population of XY SSCs exists (15). Surgical sperm procurement for males with KS is, therefore, feasible with aneuploidy rate of KS sperm found to be ~5% (16–18).

Since testicular dysfunction is a progressive decline following adolescence in patients with KS, it has been suggested that surgical retrieval may not be needed and that ejaculated sperm may possess enough sperm for cryopreservation, particularly in non-mosaic KS adolescents (19, 20). Spermatogenesis may be possible due to 47, XXY spermatogonia producing mature spermatozoa or from the presence of 46, XX spermatogonia (resulting from intratesticular mosaicism). Studies have investigated spermatogenesis in patients with KS with variable results; sperm was found in 8.4% (*n* =11) (3), in 4.2% (*n* = 2) (21), in 7.7% (*n* = 4) (19), and in 0 patients (22, 23). The presence of sperm in ejaculate in KS, therefore, appears to be a rare occurrence; however, a study by Rives et al. (24) of eight adolescent patients with KS, highlights that early exploration of fertility including semen analysis (though not always successful) is beneficial to allow patients to consider other options of FP and plan for their future. In a pediatric setting, success in finding sperm in the ejaculate for cryopreservation may depend on several factors including level of pubertal maturation, prior hormonal treatment, and genetic mosaicism. Though exact predictors of success are unclear, with a lack of data comparing outcomes in different age groups, it has been suggested that success of finding motile sperm in the ejaculate is likely to be higher in pubertal patients with KS prior to germ cell loss, hyalinisation of seminiferous tubules, and Leydig cell hyperplasia (11). Overall, there are limited studies to suggest significant success rates in finding sperm in the ejaculate for cryopreservation in the pediatric population with KS, and the quality and viability of ejaculated sperm in generating live births remain further unclear.

Surgical retrieval remains the most utilized and reliable method of retrieving sperm in patients with KS.

Conventional testicular sperm extraction (cTESE) was used prior to the late 1990s for harvesting sperm in patients with KS, where testicular parenchyma is biopsied through incisions in the tunica albuginea to obtain random seminiferous tubule specimens to search for sperm; however, this technique was disappointing, with reported SRR of only 25 to 40% (25–27). Postulating that small and isolated foci of seminiferous tubules may contain intact spermatogenesis in both patients with KS and NOA, micro-TESE has been developed. This technique involves the use of a microscope to visualize and selectively sample the most promising areas of relatively healthy seminiferous tubules, whilst minimizing trauma to testicular tissue during surgery. Significant improvements in SRRs with micro-TESE for patients with KS have been reported. The largest series to date was reported by Ramasamy et al. (28), which included 68 men with KS and SRR, pregnancy, and live birth rates of 66, 57, and 45%, respectively. A meta-analysis conducted by Fullerton et al. (29) of 373 men with KS showed the superiority of micro-TESE in terms of SRR when compared with cTESE (55 vs. 44%); however, it is important to note that although results appear to be encouraging, micro-TESE success depends highly on not only the experience of the surgeon but also the presence of a dedicated andrology and embryology team with immediate sperm examination feedback and to guide the extent of dissection.

Sperm retrieval *via* TESE in a pediatric setting was first reported in 2001 by Damani et al. (30), who successfully cryopreserved 3 vials of sperm-containing samples from a 15-year-old patient with KS. Since then, early FP has become a frequently debated issue. Those in favor of early SRR argue that spermatogonia are present in prepubertal boys, but a decline in puberty and the process accelerates during adulthood (31). Unlike NOA males with normal karyotype, levels of FSH, inhibin B, inhibin B/FSH ratio are unable to predict fertility for patients with KS (12). Early FP, therefore, theoretically provides the optimal timing for successful sperm retrieval, with younger age in patients with KS suggested being an important predictive factor (31). This is further supported by evidence of gradual loss of testicular function during puberty in patients with KS, suggesting that FP should be considered prior to this decline. In a review of 76 studies, SRR in males with KS under the age of 16 years was low at 0–20% but increased to 40–70% in those aged between 16 and 30 years (32). Bryson et al. (33) conducted a large retrospective series which found that SSR in males with KS under 30 years was significantly higher than those older than 30 years (81 vs. 33%, *p* < 0.01). Although these results appeared to be encouraging, available literature investigating FP in young patients with KS has limited sample sizes, and the reported wide range of SRRs is likely due to variation in testosterone pre-treatment, mosaicism/non-mosaicism, and level of testicular dysfunction. As such, no current guidelines exist on optimal timing for FP in KS.

On the contrary, several studies showed low or comparable SRRs in young men with KS. Plotton et al. (34) used sperm retrieval in 41 XXY men, which showed no statistically significant increase in SRR in young patients with KS (15–23 years) compared with older patients (>23 years) with rates of 52 and 62% respectively. In numerous studies investigating sperm retrieval in young men with KS (<25 years), SRRs ranged from 0 to 70% (5, 23, 35), similar to the literature of SRRs in adult patients with KS of 28–70% (36). These controversies necessitate further investigation and challenge the suggested clinical utility of early sperm retrieval (37). Another important argument against SRR in pediatric patients with KS is the potential testicular trauma as a direct result of surgical sperm retrieval procedures. Subsequent potential loss in testicular parenchyma and decline in Leydig cell function post-operatively has been a concern. A meta-analysis by Eliveld et al. (38) included 435 men with NOA and found that 85% of men returned to baseline by 1 year following surgery; however, the study also identified a statistically significant decrease in testosterone level and an increase in the risk of developing hypogonadism within 6 months after TESE in patients with KS, and time required to return to baseline testosterone level for this group of patients appeared to take longer. With a lack of statistically significant improvement in outcomes from early retrieval compared to adult KS outcomes, it could therefore be argued that FP should be explored in adulthood, where certain challenges surrounding complex ethical issues, cryopreservation, subsequent testicular damage, and psychological impacts can be avoided.

The concept of *in vitro* spermatogonial stem cell (SCC) propagation and auto-transplantation has been explored recently to provide another means of treating patients with KS and NOA. A meta-analysis (39) presented that spermatogonia were found in 46.4% of peripubertal patients who were negative for spermatozoa (compared to 24.3% in the adult group). Since the peri-pubertal period in KS is associated with the start of the loss of SCCs, this timeframe could present a potential “window” for isolating SSCs for auto-transplantation to generate spermatozoa. Its feasibility has previously been tested in a mouse model with XXY testes (40), which demonstrated its ability to perform complete spermatogenesis with human SSCs. Suggestions have been made that patients who are negative for spermatozoa at micro-TESE but positive for SSCs could be offered a procedure of *in vitro* SSC propagation and auto-transplantation as an alternative, though this research is still in its infancy and not yet translatable to the clinical setting. Furthermore, no studies have evaluated the full procedure in pediatric patients, methods are still being developed, and the appropriate harvest interval is also currently unknown. Further research into spermatogonia isolation in pediatric patients with KS and SSC propagation could provide evidence for an alternative method of FP in a subset of patients who do not have spermatozoa.

## Testosterone Replacement Therapy and Spermatogenesis

Manipulation of testosterone levels prior to FP has been suggested to benefit by optimizing spermatogenesis. To allow normal spermatogenesis to occur, appropriate signaling from the hypothalamic-pituitary-gonadal axis is fundamental. Both FSH and maintenance of high intratesticular testosterone levels (50–100 fold higher than serum) in response to LH are critical for spermatogenesis. Normally, endogenous testosterone directly inhibits GnRH and LH release at the hypothalamus and pituitary levels, leading to downstream attenuation of testosterone production (41); however, testosterone replacement can suppress spermatogenesis as it causes a reduction in the intra-testicular testosterone level and suppresses FSH level due to negative feedback on the hypothalamic-pituitary-gonadal axis (42). Therefore, one argument for pediatric FP in KS is to explore fertility potential prior to the damaging effects of testosterone replacement therapy (TRT).

Testosterone supports the development of secondary sexual characteristics, adequate bone mass, and improved muscle strength. Furthermore, testosterone therapy has been shown to have an important role in reducing the risk of cardiovascular diseases and metabolic syndrome in patients with KS (43); however, due to the lack of randomized trials on the effects of testosterone (44), no clinical guidelines on testosterone therapy exist. A recent survey of 232 specialists found that practices of testosterone therapy are variable (45), with rising gonadotropin levels, declining testosterone, and clinical symptoms of hypogonadism being the most frequent reasons for testosterone replacement initiation.

Adequate testosterone levels achieved with testosterone pre-treatment are correlated with better SRR from micro-TESE in patients with NOA (46), whilst high testosterone can contribute to impaired spermatogenesis. Various studies have investigated the use of testosterone within the context of FP in patients with KS. Plotton et al. (34) compared SRR in 41 patients with KS and found a non-significant difference in SRR between men that had and had not been previously treated with testosterone (*p* = 0.98). Alternatively, Schiff et al. (13) found that SRR was lower (20%) in men (*n* = 5) who had previously been treated with exogenous androgens and to obtain the return of spermatogenesis after testosterone, patients may require a washout and recovery period of years. It has been reported that the median time to recover normal sperm production of 20 × 10^6^ ml^−1^ sperm after discontinuation of testosterone therapy is between 3 and 6 months, with recovery in 100% of men at 24 months (47). There is, therefore, contradictory data with regards to the long-term impact of testosterone on fertility and its impact on sperm retrieval, though the common practice is not to postpone testosterone treatment in adolescent boys with KS due to its benefits within the population with KS. Of note, not all patients with KS need TRT for its current purpose, and it is also unclear what target level of testosterone will lead to the highest SRR.

Another difficulty is the timing of TRT; in a survey, optimal timing for testosterone replacement was mostly considered between 10–13 years (30%) or 14–16 years (37%) (45), though there is no recognized consensus on optimal timing for FP. The choice of the modality of administration of testosterone is further debated; the most commonly used in clinical practice being intramuscular injection or topical (48), whilst methods vary with respect to the ease of administration, ability to dose-escalate, and side effects. Lack of studies exploring these factors in pediatric patients with KS creates challenges in implementing FP strategies within a pediatric setting.

Other types of medications can be used to increase endogenous and, in particular, intratesticular testosterone production to stimulate spermatogenesis. The human chorionic gonadotropin (hCG) acts by stimulating partially functioning Leydig cells and increase testosterone production. Long-term use of hCG alone can induce spermatogenesis in a high percentage of patients but further improvement is generated with the addition of FSH. In studies performed in men with hypogonadotropic hypogonadism, trials of hCG alone (given for 14–20 months) resulted in the induction of spermatogenesis in 70% (49) and 50% (50) of patients. In patients without adequate spermatogenesis induction, FSH was added (51). Another drug that can be used is clomiphene citrate, a selective estrogen receptor modulator that blocks the negative feedback increasing of LH and FSH secretion. Hussein et al. (52) reported a 64.3% rate of sperm in the ejaculate after treatment with clomiphene citrate in men with azoospermia who had shown hypospermatogenesis or maturation arrest on biopsy. Aromatase inhibitors may also be added, as it improves spermatogenesis by decreasing the negative effect of estrogen and stimulating FSH secretion as well as increasing testosterone levels. In a study by Mehta et al. (53) 10 patients with KS, aged 14–22 years, were treated with topical testosterone replacement and aromatase inhibitor therapy for 1–5 years before surgical sperm retrieval with success in 7/10 patients (70%). Anastrozole (1 mg) was used for a period between 6 and 24 months. Last, Ramasamy et al. (28) used medical therapy with aromatase inhibitors, clomiphene or human chorionic gonadotropin before microdissection testicular sperm extraction (micro-TESE) with testicular spermatozoa successfully retrieved in 66% of men. The different types of hormonal therapies did not differentiate in terms of SRR but men with normal baseline testosterone had the best SRR of 86%.

Choice of TRT modality, administration, timing, hormone target levels, and monitoring are therefore important factors affecting FP outcomes. Further investigation into these therapies to improve spermatogenesis could be evaluated in a pediatric setting to maximize FP potential.

## Ethical Considerations

Special considerations need to be made for the ethical and psychological aspects of exploring FP in childhood. In particular, ethical dilemmas surrounding FP in pediatric patients with KS ought to be discussed with patients and parents, and risk-benefit consideration must be evaluated prior to proceeding. During counseling, it is essential to address the rights of the children to an open future, perhaps justifying parental consent to explore FP measures in children with KS to provide the best opportunity to reproduce genetically in adulthood. Pediatric patients may need multiple consultations to understand the complex issues surrounding their future fertility, as was described by Rives et al. (24) who found that patients were not initially engaged with fertility discussions.

In the UK, storage of sperm is regulated by the Human Fertilisation and Embryology Authority (HFEA), which released the HFEA Act that outlines that patients under 18 years of age are able to consent to sperm storage if they are considered competent (section 3, Paragraph 8 and 9) (54). However, fulfilling certain conditions may also permit sperm storage without expressed consent in patients under 18 years undertaking procedures that may impair fertility. These conditions include (but are not limited to) if a patient is unable to consent but a registered medical professional deems that sperm storage would be in their best interests. It is clear that the consenting process is challenging, and certain issues may need to be addressed. For example, if patients and families have opposing views, this would require counseling and further exploration. Furthermore, although a safe and well-developed procedure, cryopreservation provides no guarantee for the viability of the sample on thawing obtained from a patient. Patients with premature infertility (such as in KS) can store their sperm beyond the usual 10 year limit following the amendment of the Human Fertilisation and Embryology Act of 2008 (54). Regarding the psychological and neurophysiological aspects of KS, studies have shown a higher prevalence of anxiety, depression, and ADHD in patients with KS (55), whilst personality profile studies have indicated higher levels of neuroticism, and a significantly lower level of extraversion, agreeableness, and conscientiousness. Though there is a high prevalence of neurodevelopmental delay, memory processing and adaptive learning difficulties in children with KS (56), there is considerable variability, and each patient will thus require a personalized treatment approach.

These concerns, as well as the multi-system involvement of FP in KS, may be further addressed by utilizing a multi-disciplinary team (MDT) approach. Similar approaches have been utilized in pediatric FP in cancer patients and could be used as a foundation to build a similar model in patients with KS.

## Conclusion

There is growing evidence to support the benefit of carrying out FP in adolescent patients with KS, although controversies remain, and such practice is still in its infancy. A lack of large-scale studies is required to establish if such a practice should be recommended. Ethical dilemmas concerning FP in pediatric patients with KS remain, and discussions should be carried out with the patient and his parents in a multi-disciplinary setting to make shared decisions.

## Author Contributions

CP, AC, AG, and WL wrote sections of the manuscript. CP formulated the first draft. All authors contributed to manuscript revision, read and approved the submitted version, and conception and design of the study.

## Conflict of Interest

The authors declare that the research was conducted in the absence of any commercial or financial relationships that could be construed as a potential conflict of interest.

## Publisher's Note

All claims expressed in this article are solely those of the authors and do not necessarily represent those of their affiliated organizations, or those of the publisher, the editors and the reviewers. Any product that may be evaluated in this article, or claim that may be made by its manufacturer, is not guaranteed or endorsed by the publisher.
